# Movement as Method: Some Existential and Epistemological Reflections on Dance in the Health Humanities

**DOI:** 10.1007/s10912-021-09679-1

**Published:** 2021-03-03

**Authors:** Aimie Purser

**Affiliations:** School of Sociology & Social Policy, University of Nottingham, Nottingham, NG7 2RD UK

**Keywords:** Dance, Arts-based methods, Arts-based therapy, Body-subjectivity, Merleau-Ponty

## Abstract

The embodied creative practice of dance facilitates a particular kind of awareness or attunement which can inform both the therapeutic and the intellectual work of the Health Humanities. This paper therefore considers dance as a way of ‘doing’ Health Humanities in two interlinked ways: dance as a way of healing and dance as a way of knowing. In bringing together carnal and the creative dimensions of human experience, dance offers us a way of making sense of our place in the world that provides us both with much needed existential security (tethering) and much needed epistemological freedom (untethering).

## Introduction

The interdisciplinary approach of Health Humanities allows us to (re)locate concerns with ‘health’ in the context of broader questions about how we might understand the human condition and enhance or ameliorate human experience. This paper considers the question of how we might ‘do’ the intellectual and therapeutic work of the Health Humanities in a way that transcends the traditional dualist divisions between the Health Sciences and the Humanities and between body and mind. Dance, with its status as both embodied practice and creative art, is explored as a way of ‘doing’ Health Humanities that speaks to both the existential and the epistemological concerns that underpin it: dance as a way of healing and dance as a way of knowing. In particular, I explore the type of attunement to self, others, and world fostered in improvised dance practice and argue that dance’s capacity to allow us to reconnect with and give expression to elements of our embodied and emplaced (inter)subjectivity underpins its ability to function as a method of recovery and a method of research.

Drawing on the philosophical framework of the non-dualist work of the French Existentialist Phenomenologist Maurice Merleau-Ponty (1908-1961), I elaborate an understanding of dance which attends to both its bodily and its artistic qualities. My contention is that in bringing together the carnal and the creative dimensions of human experience, dance offers us a way of making sense of our place in the world that provides us both with much needed existential security (or tethering) and much needed epistemological freedom (or untethering). In this way it contributes to both the ameliorative and the analytical aims of the Health Humanities.

## What are the health humanities?

Although the study of human culture was originally conceived in opposition to the study of the divine, in modern universities ‘Humanities’ or ‘Arts and Humanities’ tend to serve as an umbrella term under which we might collect those whose engagement with human experience and cultural achievement is focussed through theology as well as philosophy, history, fine Art, literature, Classics and other related disciplines. The cultural and linguistic turns since the 1960s in social science scholarship have also served to develop the focus of anthropology, Sociology, Human Geography, and others through a framework that has many synergies with the Humanities. While it is an inclusive term, Humanities does, however, stand in opposition to the tradition of thought known until the nineteenth century as Natural Philosophy: that which has become modern ‘Science.’ Bio-medical scientists share the same foundational concerns as everyone else who has battled to make sense of the human condition: that we are mortal and our worldly existence is transitory; that we suffer; that to be human is to feel pleasure but also pain; and that while life is rarely without struggle we cling to it with tenacity. Yet we see a sharp distinction between the Health Sciences’ focus on an underlying mechanism that can explain the (mal)functioning of the human body and the Humanities’ focus on an underlying meaning that can explain the point or purpose of our vitality, our mortality, and our suffering.

Under this division of labour, issues of ‘health’ in the West have been largely viewed through a framework focussed more on objective and etiologically specific descriptions of ‘how’ than subjective and wide-ranging explorations of ‘why’ in relation to human experience of morbidity and mortality. This is not to say that we have not long recognised that the words of the poet or the priest can offer us solace in the face of suffering. A thorough reconnection between the mentality of the Humanities and the mentality of the health professions, however, remains a work in progress, not least because it involves a fundamental rethinking of the dualist assumptions which underpin Western thought separating the body from the mind, the physical from the metaphysical.

How are we to define the Health Humanities, then? Perhaps it is best thought of as an emerging interdisciplinary nexus that carries a history older than the disciplines from which it is formed (see, for example, Pilgrim 2016). It is an intellectual space in which we can draw on the wisdom of the arts and humanities to help broaden our understanding of our own and others’ experiences of health and ill-health and how we might provide care which speaks to the human condition at a larger level than simply treating specific ailments of the biological body (Crawford et al. 2010, 2015; Jones, Wear and Friedman 2014). Yet, more than simply a cumulative collecting of knowledge from different perspectives, its role must be to trouble and destabilise the assumption of a clear distinction between psyche (the mind or soul) and soma (the physical body) (see, for example, Fitzgerald and Callard 2016). Thus it must question the Humanities’ disavowal of the living, breathing, fleshy body (Shusterman 2006) as much as the Health Sciences’ refusal to engage seriously with the metaphysical (Pilgrim 2016).

The Humanities and the Health Sciences are both disciplinary trajectories borne of the Enlightenment commitment to knowledge and progress: the Humanities with their commitment to the achievement of human excellence and perfection through the work of the mind and the Health Sciences with their commitment to the achievement of human perfection through complete mastery of the processes of the physical body (including those of morbidity and mortality). The encounter produced at the juncture of the Health Humanities must not, however, be one which allows these separate disciplinary perspectives to simply combine their insights in a way which leaves these Enlightenment conceits intact. Rather it must force an uncomfortable and destabilising recognition of what we might call the non-rational Other for each perspective. Thus the Humanities, generally considered quite open to transcendent forms of irrationality in the form of the psychoanalytic unconscious or the creative spirit, must confront its ultimate Other in the form of the immanent, fleshy, unthinking physical body-as -object, the lump of meat. While the Health Sciences, at home with the idea of collecting rational knowledge about the physical body, are forced to confront the significance of the artistic and spiritual dimensions of human experience.

This does not mean that the Health Humanities must completely abandon the ideal of improving the human condition. Indeed it remains invested in a commitment to both the pursuit of knowledge and the amelioration of human suffering (Crawford et al. 2010, 2015; Jones, Wear and Friedman 2014). An interdisciplinary encounter of this sort must, however, push us beyond the confines of disciplinary assumptions about what constitutes progress, to engage with a more critical understanding of how these ideas have come to take shape and how they can be challenged, opened up and collaboratively rethought through a non-dualist framework.

## Dancing beyond dualism

This paper considers how movement practices such as dance can be central to the task of ‘doing’ Health Humanities with regard to both its therapeutic and its intellectual goals. Dance has not enjoyed a high status in either the Health Sciences – where its status as a creative art has undermined any scientific status, and it has only been used instrumentally as a therapeutic tool for the pursuit of physical fitness or the generation of psychic material for later verbal analysis – or the Arts and Humanities – where its status as bodily and non-linguistic has rendered it lower status than literature, poetry or even painting. I contend, however, that as both an embodied practice and a creative art, dance is perfectly positioned to (re)unite traditional concerns of the Health Sciences – the physical body – with those of the Humanities – the achievements of the human mind and creative spirit – in a way that transcends the dualism between *psyche* and *soma*. Dance in fact embodies the radical non-dualist potential of a Health Humanities framework.

Departing from the Health Sciences’ generally instrumental approach to dance and the Humanities’ tendency to treat dance as a cultural object, my interest in this paper therefore lies in exploring how dance can be conceived of within a Health Humanities framework both as a mode of (arts-based) healing and as a mode of (arts-based) knowing. I therefore consider how the embodied creative practice of dance – and I focus here particularly on improvised movement – facilitates a particular kind of awareness of or attunement to our own embodied subjectivity and to the Others and world around us. In keeping with an understanding of Health Humanities as holistic in its approach and able to transcend the traditional dualisms of Western thought, my contention here is not that we are dealing with two separate functions of dance but rather that the awareness fostered in dance is of both existential and epistemological significance.

The following discussion is grounded in and focussed through the philosophy of the existentialist phenomenologist, Maurice Merleau-Ponty. Merleau-Ponty’s work is of particular relevance to the Health Humanities because he explores questions of the nature of human-being from a non-dualist perspective which focuses on lived experience and conceptualises human subjectivity as embodied and emplaced in the world. His work therefore provides a suitable framework for thinking through questions of meaning and sense-making in relation to our embodied being-in-the-world in a way that can illuminate both the existential and the epistemological significance of engaging in creative movement practices such as improvised dance.

## Some reflections on dance as healing: e*xistential tethering*

In this section I wish to explore the power of dance to offer us understanding and thus, hopefully, solace in relation to the difficulties of the human condition. In particular, I relate this discussion to the context of contemporary Western societies where shifts towards an aging population, the dominance of chronic illness, and the dramatic increase in mental ill-health in people of all ages have been central to precipitating the call for a (re)engagement with a more holistic approach to health and health care. In this context, I suggest that the lived experience of dance is significant in its ability to bring us to an awareness of fundamental aspects of our own humanity and our place in the physical and social worlds we inhabit. Thus the embodied creative practice of dance has the capacity to provide a kind of ‘*existential tethering’* or anchoring for the individual that can form the basis from which to explore and combat contemporary existential anxieties or despair around feelings of pointlessness and isolation.

### The moving self

In order to explore fully the particularities of the awareness experienced in dance improvisation and how this is linked to fundamental questions about what it is to be human, I turn to the work of Merleau-Ponty where philosophy is borne of an attempt to understand human-being in a way which escapes the limitations of the Cartesian dualist tradition which has dominated Western philosophy (and science). A key intervention is the undermining of the primacy of the reflective mind in the constitution of any philosophical notion of self. For Descartes, the most fundamental expression of human existence is captured in the phrase ‘I think therefore I am’. Merleau-Ponty (2002), however, argues that we have a sense of self that precedes our ability to reflect on our own thinking processes. Importantly, this primary pre-reflective sense of self is embodied and emplaced; it is a sense of our bodily orientation towards and capacity to act on the world which Merleau-Ponty calls our corporeal schema. Thus before ‘I think therefore I am’ comes what is often referred to as the ‘tacit *cogito,’* a pre-reflective feeling of ‘I can’.

The location of human subjectivity in the flesh of the human body rather than in an ethereal mind is significant because it allows a non-dualist sense of self where human bodily-being is simultaneously both objecthood and subjecthood. This phenomenon is captured in the idea of being a ‘body-subject’. This also allows an escape from the Cartesian dualist model with respect to our encounter with the world. While Descartes suggests that the sense organs are mere tools which register data to be interpreted by the conscious mind, for Merleau-Ponty (2002, 1964c), perception is always already a meaningful experience of the integrated body-subject. The non-dualist concept of body-subjectivity entails that our embodied interaction with the world can be understood as purposeful and thus meaningful, even when the intentionality involved is tacit or pre-reflective, and that our sensual experience of the world can be understood as sense-making, even when it is not fully conceptualised in reflective thought.

This has implications for understanding the nature of and possibilities for our encounters with other people. Cartesian dualism locates human subjectivity in an ethereal mind that has no physical presence in the world and thus cannot be directly observed. If human subjectivity is conceived of in terms of ‘I think therefore I am’, this leads to a solipsism in which I have direct consciousness of my own subjectivity but need to interpret the Other as an object in the world. Our ability to know of or engage with the subjectivity of another person is thus always impoverished and relies on a two-step process where observation and analysis of those who look like their bodies might be commanded by human minds is followed by an additional intellectual stage of reasoning by analogy that the Other may have a similar subjectivity to my own. In contrast to this, Merleau-Ponty’s (2002, 1964b) location of human subjectivity in the physical body and his understanding of perception as direct subjective awareness rather than a rational interpretation of sense data dissolves the privilege of introspective reflection as the only way of truly knowing subjectivity and instead suggests the possibility of a direct pre-reflective perceptual awareness of the subjectivity of the Other.

In her keynote address, ‘Why is Movement Therapeutic?’ (2010), dance scholar Maxine Sheets-Johnstone returns to her earlier engagement with Merleau-Ponty in her work on ‘The Primacy of Movement’ (1999). Sheets-Johnstone furthers the argument that the most fundamental sense of our aliveness and our being-in-the-world is not in the experience of reflective thought but in the experience of bodily movement. ‘We come into the world moving, we are precisely not *stillborn*’ (2010, 2, emphasis in the original), and all of our early interactive, communicative, and learning experiences are based in our motility. Movement, she argues, is thus ‘at the core of life’ (ibid,) and is both developmentally and logically our primary mode of awareness and interaction with world. Indeed rather than thinking of the infant as pre-linguistic in line with some kind of deficit model, Sheets-Johnstone argues that linguistic competence is, for all of us, most properly understood as post-kinetic.

In the following section, I suggest that dance affords us a particular form of attunement to the embodied self as active agent in the world, to our embodied connection with others, and to those aspects of our being-in-the-world which often go unspoken or unexamined, including the emotional, sensory, spiritual and social dimensions of our embodied experiences.

### Attunement

Prior to ‘I think therefore I am’, there is, for Merleau-Ponty, the ‘I can’: a pre-reflective awareness of our embodied orientation towards and capacity for action on the world predicated on a non-dualist understanding of perception and bodily motor intentionality. Extending this insight, Sheets-Johnstone (2010) argues that it is ‘I move’: the pre-reflective sense of our own motility that makes possible the awareness of the active living self at the level of ‘I can’ or ‘I think therefore I am’. For both Sheets-Johnstone and Merleau-Ponty, then, embodied movement around and interaction with the world is tied to the most fundamental sense of self as active agent. This is significant for understanding the therapeutic value of dance because the lived experience of improvised movement is able to return us to an awareness of embodied agency. The act of dancing attunes us and allows us to explore the primacy of movement in our interactions with the world and thus reacquaints us with feelings of self-determination, intentionality and purposiveness. Furthermore, the experience of dance performatively affirms and expresses ‘a feeling of aliveness’ (Sheets-Johnstone 2010, 3).^1^ Our primary sense of self and sense of our agentic capacities are foregrounded in the dual sense of being both performatively enacted and being brought to our awareness when we engage in the embodied creative practice of dance.

Dance can therefore offer therapeutic benefits because the experience is potentially life-affirming and empowering (Sheets-Johnstone 2010, 2-3). It affirms to us our status as living, moving beings who can experience some degree of self-determination in our interaction with the world. In the contemporary world where experiences of everyday life and particularly those of mental and physical illness are often characterised and compounded by feelings of alienation and disempowerment, the practice of dance can help ground us in our basic bodily capabilities and potential for action on the world, and thus act as a basis or existential tether from which to build a sense of self-efficacy.

For Merleau-Ponty, just as the living, moving, interacting body underpins our primary sense of subjectivity, it also underpins our fundamental awareness of our existence among other subjectivities, our sense of intersubjectivity. If self is understood as bodily – as the ‘I can’ of the corporeal schema – then we can (re)conceptualise our experience of other selves as being based, first and foremost, in bodily interaction. Indeed Merleau-Ponty (2002, 1964b) describes our sense of intersubjectivity as ‘transfer of corporeal schema’: we understand the Other through a sort of empathy based on our embodied sense of their corporeal orientation towards the world.

Again, improvised dance invites an awareness and opens up space for exploration of this fundamental aspect of our being-in-the-world: our capacity for bodily or kinaesthetic empathy. This is manifest both in the connection between dancer and spectator – dance communicates through bodies, through kinaesthetic empathy, rather than through verbal language and reflective thought – and in the connection that emerges between dancers moving together. In Contact Improvisation, for example, dancers come to appreciate the Other’s movement patterns and the Other’s intentionality and subjective aspects such as mood through silent co-movement. Indeed the connection achieved when we ‘listen’ to the Other through the body in this way can be so strong that it is often spoken about as a momentary ‘blurring of the boundaries between self and other’ (Mullis 2016, 67; see also Fraleigh 1996, 57-70; Kozel, 2008, 136-160). Dance can be healing in its ability to combat isolation and alienation by grounding us in our fundamental capacity to experience a sense of intersubjective (or ‘intercorporeal’) connectedness with the Other (Purser 2017; see also Weiss 1999).

The ability of the moving dancing body to give pre-reflective expression to (and allow pre-reflective communication of) our emotions and other non-rational aspects of our being is also significant because the practice of dance reminds us and allows us to explore the intimate relationship between emotion and motion, between feelings and doings (Sheets-Johnstone 2010, 4). Dance improvisation invites an enhanced awareness of the moving body as the site of human emotions and sensuality. Through dance, we can become more attuned to the emotional and sensory backgrounds which colour our creative and our everyday experiences. Indeed as both a creative art and an embodied practice, dance can help us ‘listen to’ and communicate those dimensions of human-being that are felt by the embodied subject but cannot (easily) be put into words, including the realms of the emotions, the imagination and the spiritual (Snowber 2012, 57; see also Ramaswamy and Deslauriers 2014). This, again, can be healing in that it is helpful in providing a more grounded sense of self. It can also help us embark on a therapeutic journey by providing us with a richer understanding of the non-rational dimensions of our human experience and a richer (non-verbal) vocabulary through which to express this and engage with imaginative possibilities for change (Cancienne and Snowber, 2003, 242; Sheets-Johnstone 2010, 8-9; Snowber 2012, 56).

Engaging in the embodied creative practice of dance is thus a way of reacquainting ourselves with the ways in which ‘listening’ to and through the body can be existentially grounding and helping us to attend to a self-determining, relational and emplaced sense of selfhood. In attending to the body as the site of self, we can also use dance to explore the ways in which identity is constructed in contemporary society through the socio-cultural inscription of the body (Cancienne and Snowber 2003, 239; see also Young 1980).^2^ The intimacy of dance, the physical and emotional proximity it invites, pushes us to explore deep-seated feelings ranging from joy to ambivalence to revulsion regarding our own bodies and identities, but also those of the Other with whom we might dance. This again may be the starting point for a therapeutic journey in which we recognise the constraints of such inscription and through the creative embodied practice of dance can begin to explore our capacity for performative resistance (Mullis 2016, 68). Furthermore, the ability of dance to communicate at the level of kinaesthetic empathy can allow it to generate a direct empathic awareness within us of the embodied emotional experience of the Other. It can thus offer an experiential foundation or grounding on which to build an ethical relationship with the Other (Mullis 2016, 69), which may have therapeutic outcomes for both parties (see also O’Neill and Hubbard 2010, 50).

## Some reflections on dance as knowing: e*pistemological untethering*

In the preceding section, I have explored how the embodied creative practice of dance can offer us a sense of existential stability in a contemporary context where we often experience negative feelings of isolation, alienation, disempowerment, and purposelessness. I have argued that dance can contribute to the work of the Health Humanities as an embodied, movement-based and inherently social creative art by providing a form of existential tethering for those adrift in late modernity. In what follows, I explore dance as a method for doing the Health Humanities from a different perspective by focussing on how it might contribute to the production of knowledge about human experience.

Here I consider how the form of awareness or attunement fostered in improvised dance practice can be understood to constitute a ‘way of knowing’ – one which is based both in the living, moving, fleshy body and in the creative arts. As in the previous section, I relate this discussion specifically to contemporary Western societies, where our understanding of the human experience has been developed along dualist lines with alternative ways of knowing very much side-lined. In this context, our understanding of human suffering, both physical and psychological, is overwhelmingly framed in terms of the clinical and the biomedical, yet the existential anxieties and insecurities discussed above continue to provide a constant push to make sense of the human condition in ways that go beyond this.

I suggest that dance as a way of knowing serves an important counterpoint to the tendency to attend only to rational, cognitive ways of knowing and to the dimensions of life amenable to study through this type of framework. It allows us to attend to the uncertain, the ambiguous, and the plural. I argue that the *epistemological* significance of dance does not lie in its ability to offer (existential) surety/security but rather in its ability to *destabilise* traditional dualist divisions between body and mind, science and art, rationality and irrationality and traditional ideas of what constitutes knowledge. The use of the embodied creative practice of dance as a way of knowing in the Health Humanities thus allows for ‘a loosening of traditional epistemologies’ (O’Neill and Hubbard 2010, 47) or an ‘*epistemological untethering.*’

### The beyond-rational knower

In order to explore how dance can be considered a way of knowing, it becomes necessary to engage with the body as a site of knowledge and with artistic practice as a process of coming to know rather than simply the expression of pre-formulated ideas. The philosophy of Merleau-Ponty offers a route into these discussions through his conceptualisation of the body as a site of subjectivity and through his articulation, in his later work (1964a, 1969), of the parallel between the way the phenomenological philosopher comes to make sense of the world and the way the artist comes to make sense of the world through embodied creative practice. For Merleau-Ponty (1964a), scientific thinking, and indeed much of contemporary philosophy, has invested too much in abstracting itself from the lived reality of being-in-the-world. These trends have rendered knowledge that is something achieved through contrived experimentation and rational, objective analysis. Yet in the Western intellectual tradition’s striving for objective knowledge – the view-from-nowhere (Nagel 1986; see also Haraway 1988) – Merleau-Ponty (1964a) considers that it alienates itself from the lived experience of being-in-the-world and moves us further away from a true understanding of the nature of human-being.

In place of this, Merleau-Ponty proposes that the purpose of scientific and philosophical enquiry should not be to describe a view-from-nowhere but rather to make sense of our embodied situatedness within the world. This entails a recognition of the role of the body in positioning us as knowing – pre-reflectively perceiving, intentionally interacting, and sense-making – subjects in our physical and social environment. It is by virtue of our embodiment that we are perceptually and interactively located in and oriented towards the world; our embodied positionality gives us our point of view, the perspective from which we know (Merleau-Ponty 2002; see also Ahmed 2006). The idea of knowledge as situated and embodied has also been a central tenant of feminist epistemology (see Harding and Hintikka 1983; Lennon and Whitford 1994; Crowley and Himmelweit 1992), and this and related insights have been developed into an emerging range of research concerns and approaches in the feminist social sciences and beyond. Such work considers those pre-reflective embodied dimensions of knowledge which have generally been ignored by the Western intellectual tradition such as the sensory, the affective and the kinaesthetic (see Pink 2015; Vergunst and Ingold 2016).

In addition, there is a fast developing interest within the Humanities and the Social Sciences in arts-based research methods, emphasising the ability to of art to engage us at the levels of the emotions, the senses, the imagination and the spiritual (Leavy 2015, 2018). As Merleau-Ponty (1964a) suggests, there is grounds for thinking through how art, and in particular an embodied movement-based art form such as dance, can act as a way of knowing which could potentially move us beyond an over-investment in objectivity and the dominance of entrenched dualist traditions within disciplinary approaches. As is proper to the goal of the Health Humanities as described above, the taking seriously of dance as a way of knowing is epistemologically significant because it entails a destabilising encounter with the non-rational Other on both sides of the Health/Humanities divide. The Humanities are required to recognise the sensuous fleshy body as generative of knowledge, and the Sciences are required to recognise the creative artistic impulse as generative of knowledge. It offers us a way of ‘making sense’ that takes us beyond the stable ground of rationality and objectivity and sets us adrift amongst ambiguity and uncertainty in ways that are potentially uncomfortable but ultimately highly productive.

In the following section I continue to explore the particular forms of attunement afforded in dance but with particular emphasis on how these may constitute modes of knowledge (co-) production. That is, how they may be epistemologically significant as well as existentially significant.

### Dance as coming to know and as knowing-with

In rearticulating what it is (or might be) to do philosophy from a perspective that takes seriously the knower’s lived experience and embodiment in the world, Merleau-Ponty allows us to see the limitations of a purely rational approach and how we might expand it to account for other dimensions of knowing. Importantly for thinking through the question of how art forms such as dance can do philosophy, phenomenological philosophy suggests a model of inquiry based on an

expansive definition of philosophy [which] holds that a philosopher need not develop a specific argument – complete with a thesis, supporting evidence, and conclusion – but may provide significant insight and stimulate reflection on conceptual issues. (Mullis 2016, 60)

While Merleau-Ponty’s (1964a, 1993) own interest was in the parallel between painting and phenomenological philosophy, his insights into how the painter responds to the world through his or her embodied art-making can equally, if not better, be applied to the embodied creative practice of dance. For Merleau-Ponty, creative art is not the *re*-presentation of something that exists in the world or indeed of ideas that exist in the mind of the artist. Rather it is a process of direct, tacit or pre-reflective engagement with the world and is in-itself a process of sense-making wherein new meaning emerges, and we find ourselves surprised and enlightened by it. This locates sense-making in the living, moving, perceiving body and in the situated, embodied, sensuous, and affective interaction between artist and world. Dance can therefore be understood as a process of coming to know, and the dancing body can be understood as ‘a site of knowledge’ or ‘a locus of discovery’ (Cancienne and Snowber 2003, 237, 240; see also Snowber 2012; Snowber 2018) by virtue of its capacity to both move and be moved; its physicality, its mobility, its sensuality, and its affective expressiveness and responsiveness.

As discussed above, the particular form of awareness fostered in dance improvisation attunes us to the pre-reflective dimensions of our being-in-the-world. While aspects of human-being such as our connection with nature often feel lost to us in contemporary society (this is often experienced negatively as loss or alienation), in the embodied creative practice of dance we are able to attend to such embodied and affective connections and to the spiritual dimensions of human-being. As Snowber argues, ‘dance awakens us to emotional and spiritual intelligence’ (2012, 57). As a creative practice it involves ‘accessing places of emotion and inspiration’ (56) and constitutes a mode of embodied sensuous knowing ‘that has the capacity to connect body, mind, heart, soul and imaginative thinking’ (54). I have argued above that this process is experienced as existentially grounding – it combats feelings of alienation, for example – but my focus in this section is on how, through taking this experience seriously in epistemological terms, we can experience an untethering and let ourselves be swept off the prescribed path of rational inquiry towards an exploration of alternative forms of knowledge and ways of knowing.

The attunement to the pre-reflective is significant in this process of epistemological untethering both because it provokes reflection on those dimensions of lived experience of which we are not normally fully consciously aware, and because it allows us to explore pre-reflective emotional, sensual, spiritual, relational dimensions of our experience without pinning them down through rational thought processes. In this way, dance as a way of knowing allows us to do justice to uncertainty and ambiguity, to explore sensations and meanings in a way that doesn’t force crystallisation of ideas in the form of ‘I think this about it’ or ‘this makes me feel like this’ or ‘I am this kind of person’ and allows us to work with/through multiple, shifting, emerging meanings (Snowber 2012, 57; see also Hogan and Pink 2010).

The dominant understanding of what can count as knowledge is thus opened up when we recognise dance as a mode of inquiry to include embodied, sensuous, emotional, relational, and spiritual ways of knowing. This means that dance as a mode of knowledge production is able to offer us a more holistic form of knowledge that is freed from the constraints of rationality and objectivity and able to explore the more ambiguous and uncertain dimensions of the human condition in a non-reductive way. The Health Humanities itself emerges from a recognition that we need a way of knowing that transcends entrenched disciplinary boundaries, and dance, in bringing the body into dialogue with the Humanities and the creative impulse into dialogue with Health, is able to function as a way of knowing that can generate the truly non-dualist knowledge the Health Humanities seek.

Dance is also interesting as a way of knowing because it is performative: improvisation is an exploratory process through which meaning emerges and simultaneously, a process of the expression of that meaning in the form of dance. I have noted above that Merleau-Ponty does not agree with the dominant Cartesian assumption that self-knowledge can only be achieved through introspection and that subjectivity is essentially located in the cognitive processes of the self-aware individual, accessible to others only through a rather unsatisfactory process of deduction and analogy. Instead, for Merleau-Ponty, subjectivity is embodied, present in the world, and can be directly perceived by the Other; new meanings or ideas that occur to us emerge by virtue of our capacity for this direct dialogic interaction with the Other. Dance is exemplary of this in that it produces insight into aspects of our experience not through reflective introspection but through fostering ‘thinking in movement’ (Sheets-Johnstone 1981) where new ideas emerge and are simultaneously articulated in the dancing body during the process. Dance is also unique amongst the performing arts in that it articulates sensual, emotional, and spiritual ways of knowing through the expressive moving body in a way that decentres the verbal (and the static visual) and emphasises our capacity for interpersonal understanding at the level of kinaesthetic empathy (or transfer of corporeal schema, in Merleau-Pontian terms). It allows us to engage directly with the Other’s lived experience at the pre-reflective level.

These characteristics are significant because they relate to dance’s ability to function not only as a mode of inquiry for exploring our own experiences but also as a research method for the Health Humanities which allows engagement with experiences beyond our own. Questions of how we can access those aspects of others’ experiences and perspectives that cannot easily be put into words have been central to recent developments in social science research methods. These have focussed on exploring how we can gain an understanding of, or rather gain empathic access to, the sensory, emotional, imaginative, and spiritual backdrop to other people’s experiences; that is, how we can access their *ways of experiencing* through a mode of inquiry that attends to those dimensions which are often difficult to pin down verbally in a non-reductive and non-reifying way (Pink 2015; Hogan and Pink 2010; Thrift 1997; Thrift 2007).

Hogan and Pink (2015) have suggested that the use of art-making within the research process can be valuable for the development of empathic access to the Other’s lived experiences and interior states – to their own particular way of way of being-in the world – in a way that holds open uncertainty, ambiguity, and multiple meanings rather than reducing things to a single narrative. The sensuous intimacy of creative dance improvisation – especially but not solely when touch is involved – allows both the articulation and the intersubjective communication of these dimensions of human experience without reifying them in reflective thought. This does not mean that dance-based research in the Health Humanities must completely avoid verbalisation and reflection. Rather it allows us to think about opening up our research approaches so that acts of reflection and verbal dialogue can sit alongside pre-reflective ways of knowing and non-verbal dialogue. It is about creating a space of ‘potential’ where the tensions inherent in the encounter between rationality and the irrational dimensions of bodily and artistic knowing precipitate ‘transformative possibilities’ for those involved and thus for the development of our thinking about the human condition (O’Neill and Hubbard 2010, 47). For O’Neill and Hubbard this process of researching in a way that engages with the space (and the tensions) between rationality and irrationality and between art and social science can provide us with ‘multi-vocal dialogic texts’ which go beyond what is normally captured in scholarly writing by ‘mak[ing] visible emotional structures and inner experiences as sensuous knowledge’ (ibid).

The use of dance as a research method also draws attention to the simultaneous individual and social nature of knowledge (see also Hogan and Pink 2010, 164). If we return to Merleau-Ponty’s insight that our capacity to learn about, to know, to respond practically and intellectually to the world is based in our embodiment in that world, it clear that our position as knowers is unique to each uniquely embodied individual. If knowledge is not from a ‘god’s eye view’ or ‘view from nowhere,’ the knowledge that each of us possesses or creates is specific to us and our embodied situation in and orientation towards the world. Yet it is also the case that Merleau-Ponty’s conceptualisation of subjective knowing as embodied allows us to appreciate that knowledge is not trapped inside the isolated Cartesian mind; rather we enact our ways of knowing through our bodies and we are able to share in and connect with other’s ways of knowing by virtue of our mutual embodiment in a shared world. Dance shows us particularly clearly how interaction with the world and the ways of knowing this entails are specific to each individual living moving body – no two people’s improvised dances will be the same and we struggle to directly read off the meanings contained in dance – but also how the Other’s ways of knowing can be engaged with through co-movement and how all our ways of knowing are developed through this bodily intersubjective interaction.

Dance as a way of knowing is therefore epistemologically significant because it is both perspectival and collaborative. It opens up ways of exploring and expressing aspects of self and personal experience, but those ways of knowing are never entirely private to the individual, rather they come into being and are simultaneously communicated through the dancer’s bodily interaction with the physical and social worlds around them. The introduction of creative movement into the research process thus helps us attend to the research subject as an embodied knower actively involved in sense-making practices and to the role of the researcher as both ‘empathic witness’ to and collaborator in the production of knowledge (O’Neill and Hubbard 2010, 50).

Rather than seeking to judge or analyse the Other’s knowledge, the researcher directly empathically witnesses or enters into the Other’s way of knowing through co-movement or mutual presence in a shared space. It has been noted above that the boundary between self and other can be blurred in the moments of intense intercoporeal attunement and synchronisation that occurs when dancing with someone, yet while Merleau-Ponty’s conceptualisation of ‘transfer of corporeal schema’ allows for such moments, it is important to note that he does not suggest that two embodied individuals will meld into one subjectivity. Rather he is interested in our capacity as individuals for mutual (kinaesthetic) empathy *across* the gap between us. Importantly then, dance improvisation gives us a model for the production of knowledge which emphasises the importance of research being a process of reaching out towards and collaboratively communicating with the Other, but it is one that leaves space for multiple meanings, ambiguities and gulfs of understanding to emerge and be recognised within the research process rather than privileging rationality, instrumentalism and consensus.

## Concluding thoughts: dance, holism and humanity

This paper has elaborated the ways in which the embodied creative practice of dance is able to transcend dualist divides between body and mind and between individuality and collectivity. Dance, as a method of both research and recovery, returns us to the most fundamental aspects of our shared humanity: our mutual embodiment in a shared world and the primacy of movement in all our sense-making processes. Yet as it returns us, it also sweeps us onwards. It facilitates a reaching out across boundaries, taking us beyond our current understandings through the fostering of intersubjective empathy, and of integrated ways of knowing that include aspects of experience such as the sensory which have previously been excluded from academic writing. The carnality and the creativity of improvised dance movement thus combine to make it uniquely appropriate as a method for ‘doing’ the Health Humanities in terms of (and combining) both its recovery-oriented and its research-oriented agendas.

As I have suggested above, if the Health Humanities is to revitalise and further our understanding of health and the human condition, it must function as a space of genuine, and sometimes uncomfortable, encounter across (dualist) divides. The potential of dance as a method for this intellectual and therapeutic project lies in its capacity not only to blur boundaries – between body and mind, physicality and artistry, self and other – but also to hold open the tensions and ambiguities that emerge when we go beyond ways of knowing and ways of being (with each other) that are predicated on a notion of an atomistic (solipsistic) subject, a rational mind in encased in the body-as-object.

Dance as method allows exploration of ‘the inarticulate, sensory experiences of illness [which] often remain obscured by exclusively verbal or textual inquiry’ in both academic research and clinical practice (Eli and Kay 2015, 63). The very fact that what is experienced in dance cannot be easily or entirely satisfactorily expressed in words is significant here, not only because it suggests that there exist dimensions of embodied (sensory, emotional, and spiritual) experience beyond those we can access through purely language-based therapy or research but also because the resistance these experiences show to being ‘pinned down’ in words helps us appreciate that we are dealing here with non-rational aspects of the human condition characterised by ambiguity and plurality. Indeed as a practice that is based in both our physicality and our artistic creativity, we might think of dance as escaping the confines of rational thought twice over. As such, it is a way of knowing that can untether or free us from the constraints of the traditional epistemological frameworks of both the Humanities and the Health Sciences, whilest it is also a way of being-in-the-world which functions to tether or ground us in some of the most basic – and too often overlooked or unarticulated – aspects of our shared (fleshy) humanity.

## Endnotes

^1^ It should be noted here that in prioritising embodied agency and indeed dancing agency, this conceptualisation of human-being is not intended to reinforce an ableist understanding of physical impairment. It is not the level of mobility or the level of skill in dance that determines the quality of the experience of agency. Rather the point is that people of all different sensory and motor abilities can use some form of movement for exploratory creative experimentation and to some extent register its impact in the physical, social and sensory world they inhabit.

^2^ Further exploration of the socio-cultural inscription of the body through the lens of Existentialist Phenomenology can be found in the work of Simone de Beauvoir (1997), Franz Fanon (2008) and Iris Marion Young (2005).
